# Supplementary material for: Learning curve and surgical outcome of robotic assisted colorectal surgery with ERAS program
Source: Sci Rep. 2022 Nov 29;12:20566. doi: 10.1038/s41598-022-24665-w (PMC9709162; doi:10.1038/s41598-022-24665-w)
Supplement: Supplementary file 2 — Supplementary Information 2. [file 41598_2022_24665_MOESM2_ESM.zip › RAL Raw data-Ñ[▒K/135/OP_ñw╝╨░O▒Kñσ.pdf]

正式摘要(電子簽章：000000232601455)

請選擇...reporter

臺中榮民總醫院

Operation Note

[redacted]

- 手術日期：2021/12/18
- 手術醫師：VS 林俊余 , CR 陳瑨夫 , R 張育豪
- Anesthesia : GE
- Position : Lithotomy
- Aseptic agents : Soap

Diagnosis

- Pre-operative diagnosis : S colon tumor 15 cm FAV
- Post-operative diagnosis : Ditto.s/p robotic AR

Surgical Indication

- for curative resection and diagnosis

Operation

- 1. Robotic (XI) assisted anterior resection
  2. Intraoperative colonoscopy : (+).
  3. High ligation (+) : At IMA root. Left colic artery and IMV were also ligated
  4. Take down from the splenic flexure : (-)
  5. Robotic endoGIA (45-4.3) X2 + EEA (29mm) were used
  6. Proximal cut end: 5 cm, distal cut end: 5 cm
  7. No drainage tube was inserted
  8. Indocyanine green agent was administered for checking the blood supply status of the remaining colon
  proximal colon time: 38 sec. distal rectum 42 sec

Finding

- 1. no obvious tumor at S-colon
  2. No other gross tumor nor polyps was found
  3. There was no obvious visible liver metastasis, no visible peritoneal seeding
  4. No obvious ascites was noted
  5. Bilateral doughnuts : intact ; Air leak test: Done and no air leak was found

Procedure

- 1. General endotracheal anesthesia was induced and then the patient was placed in modified Trendelenburg lithotomy position.
  2. Preoperative antibiotics were given. A Foley catheter and nasogastric tube were placed.
  3. The abdomen was prepped and draped in standard sterile fashion.
  4. A straight incision was made over the right side of the umbilicus and a single port trocar was inserted into the peritoneal cavity by open method. Peumoperitoneum was established with CO_2 inflation.
  5. The other 8mm trocars was inserted over LUQ area of abdomen, also another one over RLQ area of abdomen. The operative findings were mentioned as above.
  6. The sigmoid colon was held by grasper and the peritoneum overlying the mesentery was scored with electrocautery.
  7. The inferior mesentery artery was identified, ligated above the left colic artery with endoclips, and transected.
  8. Dissection was done above the Gerota's fascia and toward the peritoneal attachments.
  9. The colon was mobilized from retroperitoneum overlying the sacral promontory and into the pelvis.

-
  10. Peritoneal reflection was not resected.
  11. Distal descending colon and s-colon were freely mobilized after the mesoclon was all dissected.
  12. Indocyanine green agent was administered from IV line. The blood supply of the colon was examed carefully.
  13. The distal s-colon was divided with Robotic endoGIA (45-4.3) X2.
  14. S-colon was pulled out via the single port trocar wound
  15. The proximal colon was then taken out for carefully inspection, and Indocyanine green agent was administered again from IV line, to make sure it has an excellent blood supply. The mesenteric fat was gently cleared from the terminal 2-3 mm of the bowel ends.
  16. The field was isolated with moist pad and the bowel opened and sized. It was determined that a 29 mm circular stapler could be accommodated. A purse string suture of 2-0 prolene was placed. The tumor-containing tissue was removed. Then the anvil was inserted, followed by the purse string suture. The colon was then put back to the abdominal cavity.
  17. Pneumoperitoneum was constructed again.
  18. The anal canal was irrigated with distil water and then the circular stapler was introduced through the anus and pierced through the upper area (The ventral site of the body) of the stapler line.
  19. The proximal bowel with anvil in place was engaged with the circular stapler and the stapler was then fired. Two complete donuts were obtained and the distal one was submitted to pathology for margin confirmation.
  20. The anastomosis was tested for leaks with irrigation solution and air introduced through the rectum and was found to be patent and intact.
  21. The fascia of the single port site was closed with interrupted sutures via Num.1 Vicryl.
  22. The wound was further closed with 3-O Vicryl, and followed with interrupt Nylone
  23. The patient tolerated the procedure well and was brought to the POR in stable condition.
- Estimated blood loss: minimum
- 器械數量: 正確
- 紗布數量: 正確

Image

- PAINT
  - abdomen

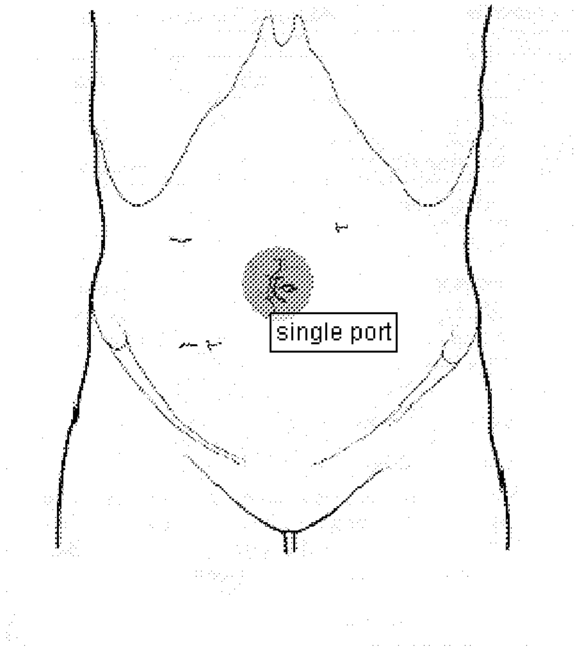

  - colon & rectum

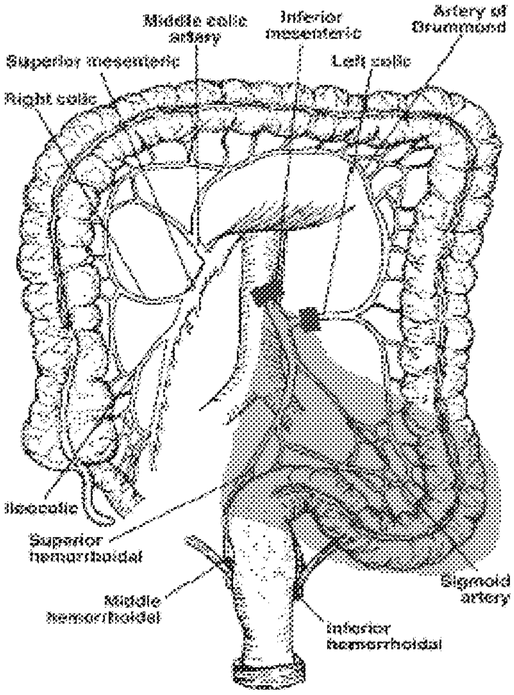

主治 2222H林俊余 / 醫師 2310K陳璿夫

正式摘要(電子簽章：**000000232601455)**

請選擇...reporter

doctorRank:B
